# Listeriosis in Pregnancy: A Rare but High-Risk Infection

**DOI:** 10.7759/cureus.47748

**Published:** 2023-10-26

**Authors:** Ana Correia de Sá, Daniela Casanova, Ana L Ferreira, Carlos Fernandes, Jorge Cotter

**Affiliations:** 1 Internal Medicine, Hospital da Senhora da Oliveira, Guimarães, PRT

**Keywords:** prematurity, fetal loss, pregnancy, bacteriemia, listeria monocytogenes

## Abstract

Listeriosis is a rare infection among the general population, with an estimated incidence in Europe of 0.49 cases per 100,000 habitants in 2021. During pregnancy, the incidence rises around ten times, peaking in the third trimester. While maternal consequences are usually mild, the potential for severe fetal and neonatal outcomes exists, leading to fetal loss, prematurity, neonatal sepsis, meningitis, and mortality. In the newborn, the clinical presentation and outcomes are associated with both gestational timing of infection and birth gestational age. We report a case of a pregnant woman with fever and nonspecific symptoms during the second trimester, leading to the diagnosis of Listeria bacteremia. We describe the steps for diagnostics, evolution, and complications and the importance of the differential diagnosis when evaluating pregnant patients.

## Introduction

*Listeria monocytogenes* is found in processed and prepared foods, and listeriosis is linked to elevated morbidity and mortality [1,2]. In Europe, it is rare among the general population, with an incidence of 0.49 cases per 100,000 habitants in 2021, and even rarer in Portugal, with no reported cases in 2021. The USA has an incidence rate of up to 0.6 cases per 100,000 inhabitants. Pregnant women are at high risk for listeriosis, with the incidence increasing by around ten times, peaking in the third trimester. This emphasizes the importance of dietary restrictions during pregnancy. Symptoms are commonly nonspecific, and consequently, diagnosis is difficult [1]. While maternal consequences are usually mild, the potential for severe fetal and neonatal outcomes exists [3].

## Case presentation

A 30-year-old pregnant woman in the second trimester (23 weeks + 4 days) presented in the emergency department with fever (maximum of 38,9°C) in the last three days, vomits, abdominal pain in the epigastric and upper right quadrant regions, and choluria within the last 24 hours. She had no headache, syncope, seizures, or any respiratory and genitourinary issues. Her medical history included class III obesity.

On examination, she had jaundice, fever of 38°C, a blood pressure of 110/64 mmHg, and a cardiac frequency of 77 bpm. Her abdomen was soft, with pain on palpation in the upper right quadrant, without rebound or guarding. Blood analysis highlighted direct hyperbilirubinemia, elevated transaminases, and an elevated C-reactive protein level (Table 1). Abdominal ultrasonography was reported as normal, with specific reference to the liver, gallbladder and appendix.

**Table 1:** Main laboratory test results * WCC - White-cell count | CRP - C-reactive protein |LDH - Lactate dehydrogenase | TB - Total bilirubin | DB - Direct bilirubin | AST - Aspartate aminotransferase | ALT - Alanine aminotransferase | GGT - Gamma-glutamyl transferase | AP - Alkaline phosphatase | PT - Prothrombin time | INR - International Normalized Ratio |APTT - Activated Partial Thromboplastin Time. ** 9^th^ day of ampicillin; *** First week after discharge; **** Second week after discharge

Variable *	Admission	5^th^ day	11^th^ day **	25^th^ day ***	35^th^ day ****	Reference value
Hemoglobin (g/dL)	12.5	10.3	10.7	12.5	11.9	12.0 - 16.0
WCC (per µL)	5.9 x10^3^	8.0 x10^3^	13.1 x10^3^	18.1 x10^3^	18.3 x10^3^	4.8 - 10.8 x 10^3^
Neutrophils (per µL)	5.1 x 10^3^	6.9 x 10^3^	5.9 x 10^3^	10.8 x 10^3^	11.2 x 10^3^	1.8 - 7.7 x10^3^
Eosinophils (per µL)	0.0 x 10^3^	0.0 x 10^3^	0.0 x 10^3^	0.3 x 10^3^	0.2 x 10^3^	0.0 - 0.49 x10^3^
Basophils (per µL)	0.3 x 10^3^	0.0 x 10^3^	0.1 x 10^3^	0.4 x 10^3^	0.1 x 10^3^	0.0 - 0.1 x10^3^
Lymphocytes (per µL)	0.4 x 10^3^	0.8 x 10^3^	5.9 x 10^3^	6.1 x 10^3^	5.7 x 10^3^	1.0 - 4.8 x10^3^
Monocytes (per µL)	3.9 x 10^3^	0.2 x 10^3^	1.1 x 10^3^	0.9 x 10^3^	1.0 x 10^3^	0.12 - 0.8 x10^3^
Platelets (per µL)	213 x 10^3^	88 x 10^3^	320 x 10^3^	330 x 10^3^	288 x 10^3^	150 - 350 x10^3^
CRP (mg/liter)	143.6	225.2	18.7	17.1	11.5	<3.0
Urea (mg/dL)	18	42	11	14	17	74 - 106
Creatinine (mg/dL)	0.67	0.88	0.51	0.61	0.51	0.57 - 1.11
Sodium (mEq/liter)	138	-	139	-	-	135 - 146
Potassium (mEq/liter)	3.65	-	4.18	-	-	3.5 - 5.1
LDH (UI/liter)	494	677	381	228	206	84 - 246
TB (mg/dL)	4.14	7.5	2.94	1.6	1.1	0.2 - 1.0
DB (mg/dL)	2.20	5.4	2.05	0.9	0.5	0.0 - 0.3
AST (UI/liter)	258	504	363	169	63	12 - 40
ALT (UI/liter)	179	352	281	255	78	7 - 40
GGT (UI/liter)	39	30	43	22	16	0 - 38
AP (UI/liter)	94	90	226	156	109	46 - 116
Albumin (g/dL)	4.1	2.8	3.6	4.1	-	30 - 118
Amylase (UI/liter)	40	-	-	-	-	30 - 118
Lipase	24	-	-	-	-	12 - 53
PT (sec.)	14.1	14.8	11.1	11.9	-	11.5
INR	1.2	1.3	1.0	1.0	-	-
APTT (sec.)	24.4	27.8	31.7	30.2	-	29.1
Fibrinogen (mg/dL)	-	319	-	-	-	150 - 400

Obstetrics consultation ruled out obstetric pathology. Physical evaluation and vaginal speculum exam were reported as normal. The fundal exam didn't reveal tenderness, and fetal and vaginal ultrasonography were reported as normal. The patient was admitted to the Internal Medicine Department and prescribed 1g/day of ceftriaxone, 1g 8/8h of paracetamol if fever, and 40mg of subcutaneous venous thrombosis prophylaxis.

On the first day of hospitalization, the patient had a remission of abdominal pain. Vomits and fever persisted with two to three spikes a day. The patient was apyretic after the third day, but on the fifth day, there was a deterioration in laboratory test results. Both transaminases and bilirubin were still increasing (Table 1), and the protein/creatinine ratio was 0.86. The blood pressure remained normal. A second abdominal ultrasonography did not reveal any abnormalities. *Listeria monocytogenes* was isolated in blood cultures and confirmed by a polymerase chain reaction (PCR) panel. Antibiotic therapy was changed to 2g intravenous ampicillin, 4/4h for 14 days. Upon review of the epidemiological context, the pregnant reported the ingestion of uncooked processed meats.

Obstetrics consultation was obtained concerning potential fetal complications. Reassessment with physical evaluation and fetal and vaginal ultrasonography was reported as normal. The association of gentamicin was considered, but dismissed due to potential fetal ototoxicity and nephrotoxicity risks and uncertain synergy with ampicillin. 

During the etiological investigation, hemolysis was documented. The initial haptoglobin measurement was decreased (<7,1 mg/dL) without a decrease in hemoglobin levels. Direct and indirect antiglobulin tests were negative, and peripheral blood smear was reported as normal. The test for glucose-6-phosphate dehydrogenase deficiency was negative. Thrombocytopenia was also observed. The blood pressure was always normal, and no proteinuria was documented. A HELLP syndrome (hemolysis, elevated liver enzymes, and low platelet count) was considered, but this hemolysis was interpreted in a non-immune context, likely attributed to the infection, given that it is one of the main characteristics of *Listeria monocytogenes* virulence.

Over the next eight days of treatment with ampicillin, the patient´s condition improved, with sustained apyrexy, decreasing transaminases levels, and decreasing cholestasis markers. However, by day nine of treatment, transaminases, and cholestasis markers worsened (increased to aspartate aminotransferase of 363 UI/liter, alanine aminotransferase of 281 UI/liter, and alkaline phosphatase 226 UI/liter), while bilirubin levels continued to decrease, suggesting probable hepatic toxicity due to ampicillin. Antibiotic therapy was switched to 480mg of intravenous trimethoprim-sulfamethoxazole every six hours for the remaining treatment duration.

The patient´s clinical condition continued to improve while transaminases started to decrease. Blood cultures set collected on the 10th day of targeted antibiotic therapy were negative, and the treatment was completed within 14 days of targeted antibiotic therapy. A second haptoglobin measurement conducted at the end of the hospitalization showed normalization. The patient was discharged asymptomatic 18 days after admission. Reinforcement of dietary restrictions during pregnancy was provided upon discharge.

A follow-up Internal Medicine evaluation in the first and second weeks after discharge indicates sustained improvement in transaminase levels and normalization of bilirubin (Table 1). The patient continued to be followed up by obstetrics appointments due to her class III obesity. Her pregnancy proceeded without complications with appropriate fetal development. At the time of labor, transaminase levels and bilirubin were normal. The woman delivered spontaneously into labor at 37 weeks by cephalic eutocic delivery, delivering a live female newborn weighing 2740g, with Apgar scores of 8/9/10 at 1st, 5th, and 10th minutes, respectively. The newborn exhibited a favorable evolution within the first week of life and during the following three months, without complications up to the current point.

## Discussion

In Portugal, listeriosis is considered a public health problem and is legally mandatory to be reported as a foodborne infection. *Listeria monocytogenes* is found in processed and prepared foods, and listeriosis is linked to elevated morbidity and mortality [1,2]. Pregnant women are at high risk for listeriosis, with the incidence increasing by around 10 times, peaking in the third trimester. This emphasizes the importance of dietary restrictions during pregnancy, like frozen and ready-to-eat meals including salads. Symptoms are commonly nonspecific, and consequently, diagnosis is difficult [1].

While maternal consequences are usually mild, the potential for severe fetal and neonatal outcomes exists, leading to fetal loss, prematurity, neonatal sepsis, meningitis, and mortality [3]. In the newborn, the clinical presentation and outcomes are associated with the timing of maternal disease onset during pregnancy and the gestational age at birth. Listeria can be transmitted to the fetus either through the placenta, via the maternal bloodstream, or directly through ascending from a colonized vaginal canal [1,4]. Listeriosis during pregnancy has a worse outcome when it affects the fetus in the early stages of gestation, often leading to spontaneous abortion or stillbirth. In later stages, preterm birth is a frequent outcome, with the highest mortality rates observed among infants born at the earliest gestational ages. Neonatal listeriosis has an incidence of approximately 8.6 cases per 100,000 live births, and it is one of the leading causes of neonatal meningitis. Symptoms occur, on average, 36 hours after birth [1]. Prompt identification and intervention are linked to better outcomes, and in cases of a high epidemiological risk of Listeria infection, empirical administration of antibiotics should be initiated before culture results are available. In pregnancy, the recommended first-line treatment involves a prolonged course of high-dose ampicillin (≥ 6g/day) for 14 days. Alternatively, in the second trimester, 10 to 20 mg/kg of intravenous trimethoprim-sulfamethoxazole every six hours, could be used. While there have been claims of synergy between ampicillin and gentamicin, this has been confirmed only in a laboratory setting [1,3].

Although this is a rare case in Europe, we report it to emphasize the challenges posed by listeriosis during pregnancy, from clinic suspicion to management after diagnosis confirmation and considerations in treatment strategy involving antibiotic choices and fetal implications. Differential diagnoses are crucial. HELLP syndrome, a more prevalent diagnosis, was considered due to hemolysis, elevated hepatic enzymes, and thrombocytopenia but was dismissed. This decision was based on the fact that these are characteristics of *Listeria monocytogenes* virulence, and the patient's blood pressure was normal with no documented proteinuria. The successful management of the patient's condition underscores the importance of prompt and adaptable interventions guided by both clinical expertise and emerging data, given the potentially harmful impact of a missed diagnosis.

## Conclusions

Bacteremia by *Listeria monocytogenes* is a rare infection with favorable outcomes in the general population but potentially severe fetal and newborn complications. During pregnancy, fever and nonspecific symptoms should be investigated, given the higher prevalence of infectious diseases. Epidemiological context and a comprehensive etiological study should be performed, given the broad differential diagnosis and potentially harmful impact of a missed diagnosis.

## References

[1] Lamont RF, Sobel J, Mazaki-Tovi S (2011). Listeriosis in human pregnancy: a systematic review. J Perinat Med.

[2] (2022). The European Union One Health 2021 Zoonoses Report. EFSA J.

[3] (2014). Committee opinion no. 614: management of pregnant women with presumptive exposure to Listeria monocytogenes. Obstet Gynecol.

[4] Mylonakis E, Paliou M, Hohmann EL, Calderwood SB, Wing EJ (2002). Listeriosis during pregnancy: a case series and review of 222 cases. Medicine (Baltimore).

